# A Strategy to Employ *Clitoria ternatea* as a Prospective Brain Drug Confronting Monoamine Oxidase (MAO) Against Neurodegenerative Diseases and Depression

**DOI:** 10.1007/s13659-015-0079-x

**Published:** 2015-12-14

**Authors:** A. Anita Margret, T. Nargis Begum, S. Parthasarathy, S. Suvaithenamudhan

**Affiliations:** Department of Biotechnology and Bioinformatics, Bishop Heber College, Tiruchirappalli, 620017 India; Department of Biotechnology, Jamal Mohamed College, Tiruchirappalli, 620020 India; Department of Bioinformatics, School of Life Sciences, Bharathidasan University, Tiruchirappalli, 620024 India

**Keywords:** (Z)-9,17-Octadecadienal, Kaempferol-3-monoglucoside, Monoamine oxidase, *Clitoria ternatea*, Molecular docking, Ayurvedic medicine

## Abstract

**Abstract:**

Ayurveda is a renowned traditional medicine practiced in India from ancient times and *Clitoria ternatea* is one such prospective medicinal herb incorporated as an essential constituent in a brain tonic called as medhya rasayan for treating neurological disorders. This work emphasises the significance of the plant as a brain drug there by upholding Indian medicine. The phytochemicals from the root extract were extricated using gas chromatography–mass spectrometry assay and molecular docking against the protein Monoamine oxidase was performed with four potential compounds along with four reference compounds of the plant. This persuades the prospect of *C. ternatea* as a remedy for neurodegenerative diseases and depression. The in silico assay enumerates that a major compound (*Z*)-9,17-octadecadienal obtained from the chromatogram with a elevated retention time of 32.99 furnished a minimum binding affinity energy value of −6.5 kcal/mol against monoamine oxidase (MAO-A). The interactions with the amino acid residues ALA 68, TYR 60 and TYR 69 were analogous to the reference compound kaempferol-3-monoglucoside with a least score of −13.90/−12.95 kcal/mol against the isoforms (MAO) A and B. This study fortifies the phytocompounds of *C. ternatea* as MAO-inhibitors and to acquire a pharmaceutical approach in rejuvenating Ayurvedic medicine.

**Graphical Abstract:**

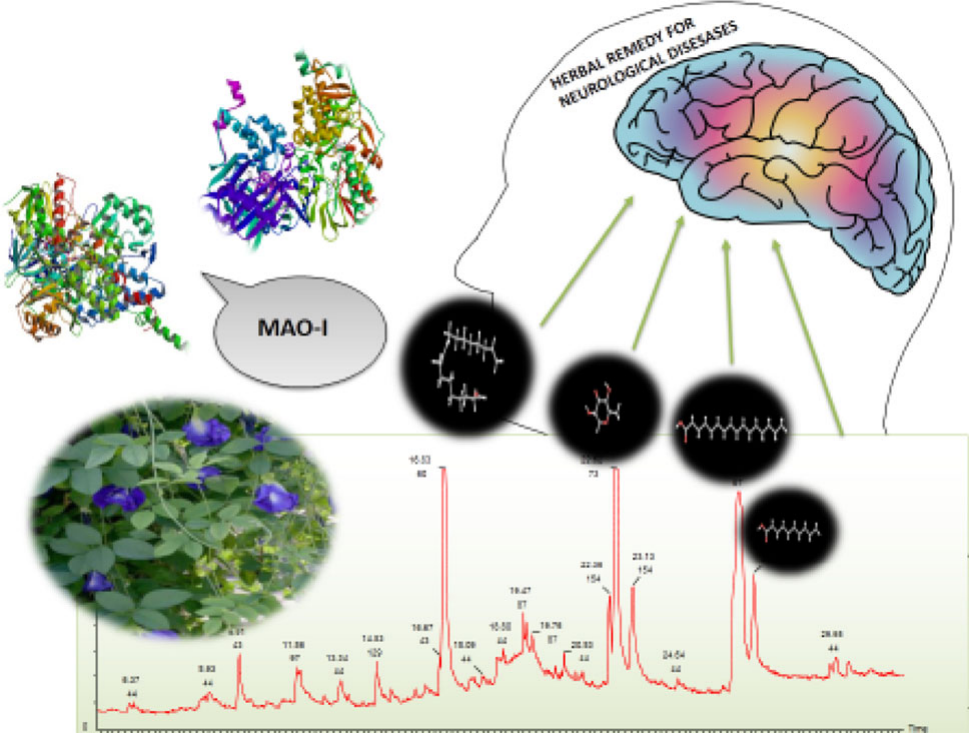

## Introduction

Alternative medicines disburse a new stratagem in restoring health and traditional medicines like Ayurveda has an embedded background and its patrons are drawn from ancient communities. The antiquity seized by ayurvedic medicine has formulated treatises to enhance mental health that promote memory and intelligence [1]. Medhya drugs constitute an assemblage of medicinal herbs that acts on the nervous system and improve mental abilities [2]. Medhya rasayana is a brain and nervine tonic that has been used for centuries in rejuvenating neurons and treating neurological diseases [3, 4]. Shankhpushpi is a reputed drug constituted in medhya rasayana whose name is derived from Sanskrit language. It illustrates a plant with flowers shaped like a conch or shankha which is a sacred instrument used in ritual worships. According to the pharmacopoeia of India, *Convolvulus pluricaulis* (Convolvulaceae) as a whole plant rightfully claim the name of Shankapushpi but, Ayurvedic practitioners have used three other medicinal herbs such as *Evolvulus alsinoides* Linn. (Convolvulaceae)*, Canscora decussata* Schult. (Gentianaceae) and *Clitoria ternatea* Linn. (Papilionaceae) [5–9].

Though all the four plants are catalogued under the same class *Magnoliopsida*, *C. ternatea* separates itself at the level of sub-class belonging to *Rosidae*, while the other three belong to *Asteridae*. *Clitoria ternatea,* is distinct among other herbs and has the property of being a good brain drug [10]. Therefore there is a sturdy instinct to evaluate the phytoconstituents of the plant to treat mental disorders. A wide range of phytocompounds including ternatins, alkaloids, flavonoids, saponins, tannins, carbohydrates, proteins, resins, starch, taraxerol, taraxerone and secondary metabolites such as triterpenoids, flavonol glycosides, anthocyanins, steroid elevates the hope of endorsing *C. ternatea* as an efficient botanical medicine combating neurological ailments. This study exonerates the phytocompounds present in the root extract of the plant with a gas chromatography–mass spectrometry assay pursued by a molecular docking against a flavoenzyme Monoamine oxidase (MAO).

This protein is responsible for the oxidative deamination of neurotransmitter and dietary amines [11–13]. This enzyme degrades neurotransmitters such as serotonin and dopamine in the brain which is coded for by the MAOA gene [14–16]. Neurotransmitters play a pivotal role in mood, arousal, and emotions, even affecting impulse control.

The isoforms of Monoamine Oxidase (A and B) are categorised based on their substrate preference and inhibitor selectivity. Inhibitors of MAO-A are clinically used as antidepressants and anxiolytics [17, 18] while MAO-B inhibitors are used for the treatment of Parkinson’s disease and for symptoms associated with Alzheimer’s disease [19, 20].

Although several synthetic monoamine oxidase inhibitor (MAOI) have emerged as antidepressant drugs, the desire of herbal medicine is excessive. They are proficient in surpassing the adverse effects and improves a better sustainability. Hence a substantial study establishing *C. ternatea* as monoamine oxidase inhibitor (MOAI) fetches a stoppage solution against depression and neurological problems which hoists Ayurveda extensively.

## Results and Discussion

Phytochemical assay of the plant *C. ternatea* was performed to divulge the essential phytocompounds which draw a base line in accessing their medicinal significance. The roots of the plant have an extended antiquity to promote mental power memory retention and alleviate psychotic stress [21]. Studies have revealed that aqueous root extract of *C. ternatea* enhances memory in rats while, alcoholic extracts of aerial and root parts of *C. ternatea* attenuated electroshock-induced amnesia [22, 23]. Hence this study determined root as the vital part that reveals the utmost essential phytocompounds.

### Extraction of Volatile Phytochemicals Augmenting Brain Function by GC–MS Assay

**Fig. 1:**
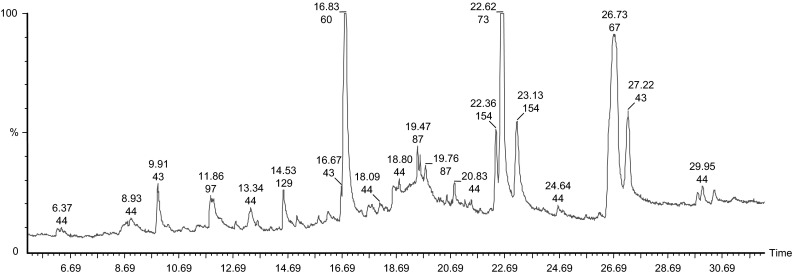
A chromatogram illustrating the presence of bioactive phytochemicals

GC–MS chromatogram analysis pertained from the ethanol extract of *C. ternatea* (Fig. 1) extricated twenty-five different compounds illustrated with twenty intense peaks indicating the presence of these phytochemical in a high constituent. The phytocompounds were detected and catalogued in parallel to the NIST library (Table 1). Among them, the most prevailing compounds are n-hexadecanoic acid (21.32 %) and (Z)-9,17-Octadecadienal (28.76 %), with a retention time of 22.62 and 26.73 min. d-Allose (17.53 %), pyrrolo[1,2-a]pyrazine-1,4-dione (5.5 %), and 2,3-dihydro-3,5-dihydroxy-6-methyl-4H-pyran-4-one (3.76 %) pursue the former compounds with a retention time of 16.83, 23.13 and 9.91 min. The foremost compound (Z)-9,17-octadecadienal is categorized as aldehyde and commonly called as linolenic acid. It is an essential omega-3 fatty acid that has an explicit therapeutic value in regulating cholesterol level in blood. Reports unveil that they have neuroprotective properties and increased intake of α-linolenic acid reduced depressive symptoms thereby maintaining robust mental health mental health [24–26]. Furthermore, palmitic acid (n-hexadecanoic acid) is one another indigenous compound which has an antioxidant property and acts as a 5-alpha reductase inhibitor [27]. Studies report that it has anticonvulsant and antidepressant property. The scavenging and inhibition of free radicals, inhibits the neurotoxicity of amyloid-β thereby offering protection against hypoxic challenges [28–30]. A diverse range of flavonoids occurs in traditional medicine that exert as sedatives and carry out anxiolytic effects. This is due to the cognitive enhancement of the up regulation of cholinergic that results in the binding of GABAA receptors [29, 30]. This results in the inhibition of monoamine oxidase thereby raising the level of noradrenalin [31]. 2,3-Dihydro-3,5-dihydroxy-6-methyl-4H-pyran-4-one is characterized as a fragment of flavanoids which when synthesised by plants, are termed as phytoestrogens. Epidemiological studies suggests that they have the potentials of treating neurologic disorders such as dementia and Alzheimer’s disease [32, 33]. Hence the analysis of GC–MS further extends a need of study to assay the antidepressant and neuroprotective aspect of the major compounds extricated from the root extract.

**Table 1:** List of extricated compounds attained from GC–MS Assay

S. no.	Peak name	Retention time	Peak area	% Peak area
1.	Name: 2-Furancarboxaldehyde, 5-methyl- Formula: C_6_H_6_O_2_ MW: 110	6.23	1485494	0.4220
2.	Name: 2,4-Dihydroxy-2,5-dimethyl-3(2H)-furan-3-one Formula: C_6_H_8_O_4_ MW: 144	6.37	1298819	0.3689
3.	Name: Pyrrolidine, 3-methyl- Formula: C_5_H_11_N MW: 85	8.93	2331724	0.6623
4.	Name: 4H-Pyran-4-one, 2,3-dihydro-3,5-dihydroxy-6-methyl- Formula: C_6_H_8_O_4_ MW: 144	9.91	13264328	3.767
5.	Name: Uracil Formula: C_4_H_4_N_2_O_2_ MW: 112	11.39	2594699	0.7370
6.	Name: Cyclohexanone,2-isopropyl-2,5-dimethyl- Formula: C_11_H_20_O MW: 168	11.85	3496772	0.9933
7.	Name: 2-Methoxy-4-vinylphenol Formula: C_9_H_10_O_2_ MW: 150	12.78	1121110	0.3185
8.	Name: Valeric acid, 2,3-epoxy-3,4-dimethyl-, tert-butyl ester, cis- Formula: C_11_H_20_O_3_ MW: 200	13.34	8129658	2.309
9.	Name: n-Decanoic acid Formula: C_10_H_20_O_2_ MW: 172	13.58	1971583	0.5600
10.	Name: 2-Butenoic acid, 4,4-dimethoxy-, methyl ester Formula: C_7_H_12_O_4_ MW: 160	14.53	6979632	1.982
11.	Name: Phenol, 2-methoxy-4-(1-propenyl)-, (E)- Formula: C_10_H_12_O_2_ MW: 164	15.04	1273167	0.3616
12.	Name: 5-n-Propylhydantoin Formula: C_6_H_10_N_2_O_2_ MW: 142	16.19	731662	0.2078
13.	Name: d-Allose Formula: C_6_H_12_O_6_ MW: 180	16.83	61745524	17.53
14.	Name: Benzoic acid, 4-hydroxy-3-methoxy- Formula: C_8_H_8_O_4_ MW: 168	17.66	1914401	0.5438
15.	Name: 1,6-Anhydro-á-d-glucofuranose Formula: C_6_H_10_O_5_ MW: 162	18.58	1833479	0.5208
16.	Name: Phenol, 2,6-dimethoxy-4-(2-propenyl)- Formula: C_11_H_14_O_3_ MW: 194	18.80	829054	0.2355
17.	Name: Tetradecanoic acid Formula: C_14_H_28_O_2_ MW: 228	19.47	2008953	0.5706
18.	Name: Phenol, 4-(3-hydroxy-1-propenyl)-2-methoxy- Formula: C_10_H_12_O_3_ MW: 180	19.76	2250140	0.6392
19.	Name: 1,13-Tetradecadien-3-one Formula: C_14_H_24_O MW: 208	20.55	1048599	0.2979
20.	Name: 1,6-Hexanediamine, 2,2,4-trimethyl- Formula: C_9_H_22_N_2_ MW: 158	20.82	1607754	0.4567
21.	Name: Pyrrolo[1,2-a]pyrazine-1,4-dione, hexahydro-3-(2-methylpropyl)- Formula: C_11_H_18_N_2_O_2_ MW: 210	22.36	13352407	3.792
22.	Name: n-Hexadecanoic acid Formula: C_16_H_32_O_2_ MW: 256	22.62	75068920	21.32
23.	Name: Pyrrolo[1,2-a]pyrazine-1,4-dione, hexahydro-3-(2-methylpropyl)- Formula: C_11_H_18_N_2_O_2_ MW: 210	23.13	19559404	5.555
24.	Name: 9,17-Octadecadienal, (Z)- Formula: C_18_H_32_O MW: 264	26.73	101281232	28.76
25.	Name: Octadecanoic acid Formula: C_18_H_36_O_2_ MW: 284	27.22	22540484	6.402
26.	Name: 13-Octadecenal, (Z)- Formula: C_18_H_34_O MW: 266	29.77	2328958	0.6615

### Molecular Docking Studies

**Fig. 2:**
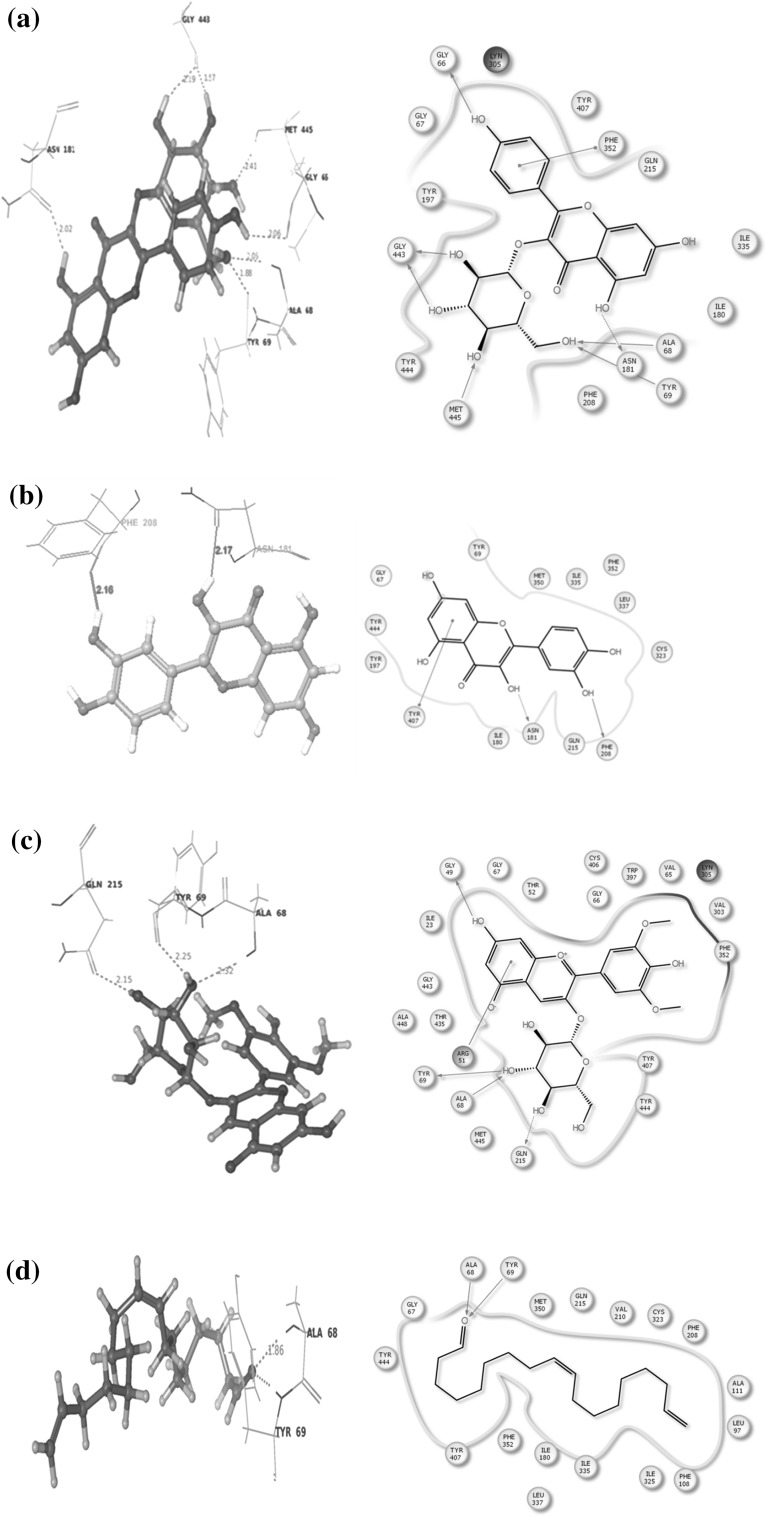

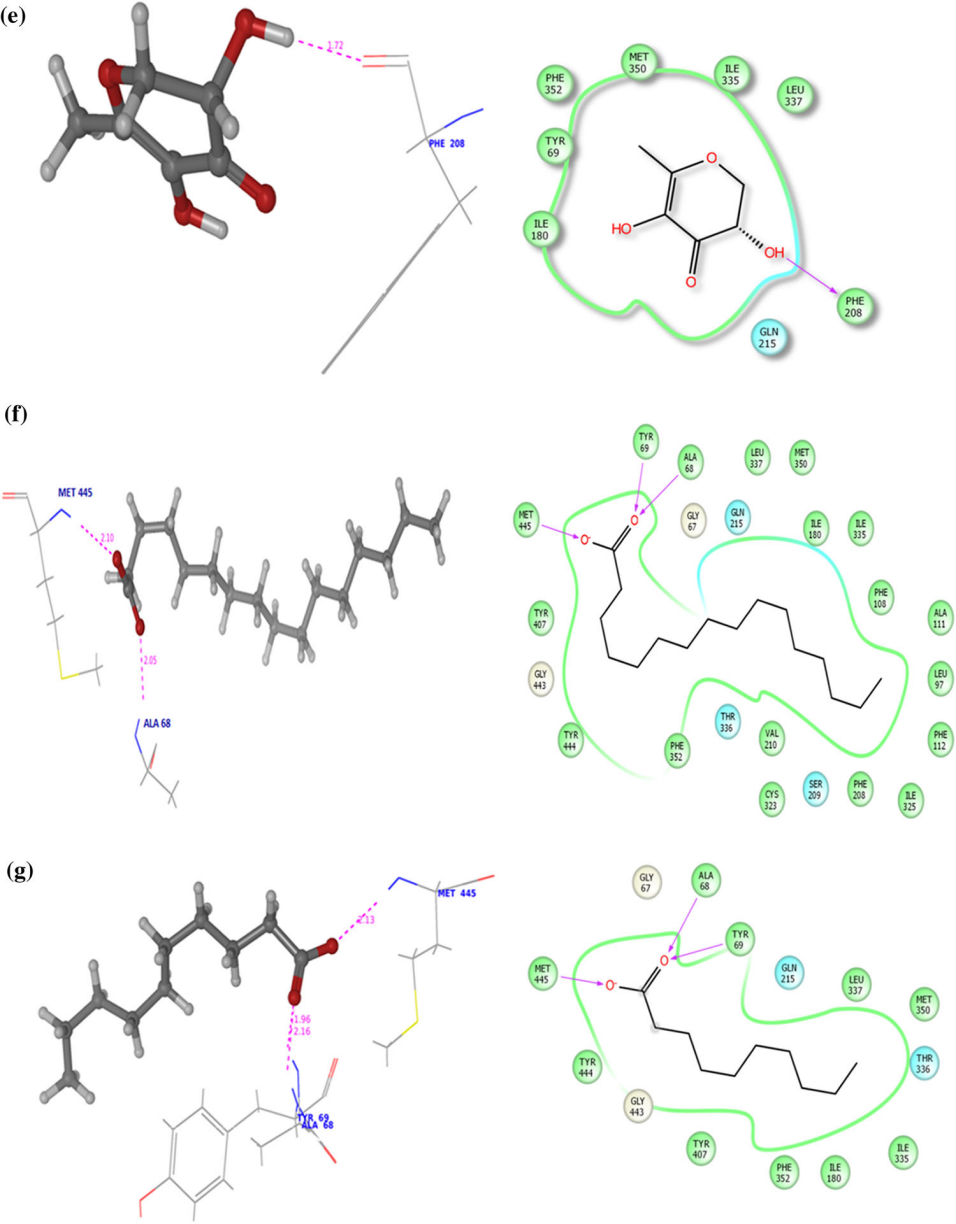
**a** Interaction between kaempferol-3-monoglucoside and Mono Amine Oxidase A conducted by the amino acid residues ALA68, GLN443, GLY66, MET445, TYR69 and ASN181. **b** Interaction between Quercetin and Mono Amine Oxidase A conducted by the amino acid residues ASN181 and PHE 208. **c** Interaction between Malvidin-3-0-glucoside and Mono Amine Oxidase A conducted by the amino acid residues ALA68, TYR69 and GLN215. **d** Interaction between (*Z*)-9,17-octadecadienal and monoamine oxidase A conducted by the amino acid residues ALA68 and TYR69. **e** Interaction between 2,3-dihydro-3,5-dihydro-6-methyl-4H-pyran-4-one and monoamine oxidase A conducted by the amino acid residue PHE 208. **f** Interaction between n-hexadecanoic acid and monoamine oxidase A conducted by the amino acid residues ALA68 and MET 445. **g** Interaction between n-decanoic acid and monoamine oxidase A conducted by the amino acid residues ALA68, MET 445 and TYR69

**Fig. 3:**
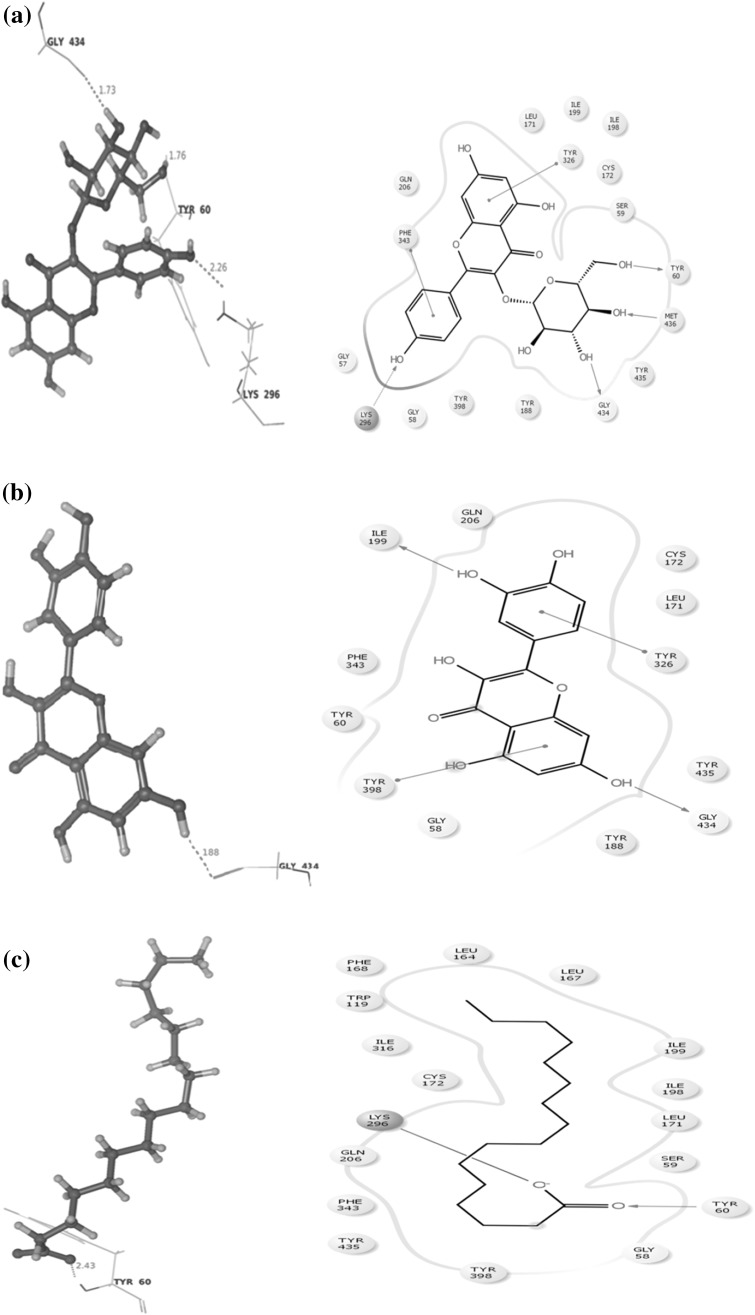

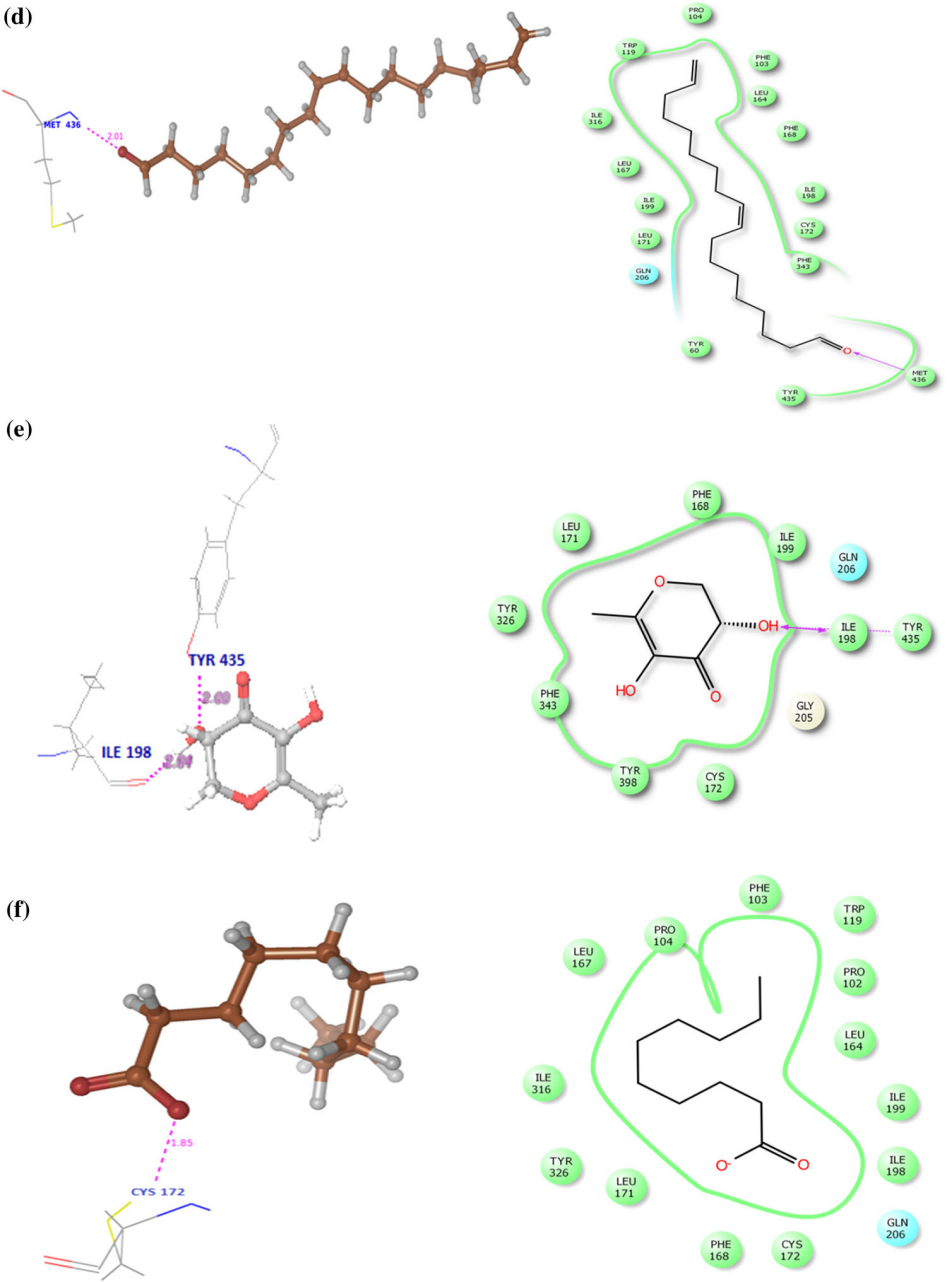
**a** Interaction between kaempferol-3-monoglucoside and monoamine oxidase B conducted by the amino acid residues LYS296, TYR60 and GLN434. **b** Interaction between quercetin and monoamine oxidase B conducted by the amino acid residue GLN434. **c** Interaction between n-hexadecanoic acid and monoamine oxidase B conducted by the amino acid residueTYR 60. **d** Interaction between (*Z*)-9,17-octadecadienal and monoamine oxidase B conducted by the amino acid residue MET 436. **e** Interaction between 2,3-dihydro-3,5-dihydroxy-6-methyl-4H-pyran-4-one and monoamine oxidase B conducted by the amino acid residue TYR 435 and ILE 198. **f** Interaction between n-decanoic acid and monoamine oxidase B conducted by the amino acid residue CYS 172

An in silico assay was done to determine the best compound by docking against the depression and neurodegenerative inducing protein MOA (Mono Amine Oxidase). Alterations in monoaminergic transmission are reported to be related with the instigation of neurodegenerative diseases such as Parkinson’s, Alzheimer’s diseases and psychiatric disorders such as depression and anxiety [34]. In the present study, four phytocompounds confiscated from the root extracts of *C. ternatea* were docked computationally into the active site of the monoamine oxidase isoforms (MAO-A and MAO-B) and were investigated to endorse their inhibitory potency. Former studies deem to have the presence of imperative primary and secondary metabolites from the whole plant that are medically significant. Combined chemical analysis have isolated many vital phytocompounds such as kaempferol (kaempferol 3-neohesperidoside, kaempferol 3-rutinoside, kaempferol 3-glucoside), quercetin (quercetin 3-2 G-rhamnosyl rutinoside, quercetin 3-neohesperidoside, Quercetin 3-rutinoside, quercetin 3-glucoside), Myricetin 3-neohesperidoside, Myricetin 3-rutinoside, Myricetin 3-glucoside and delphinidin 3,3,5-triglucoside [35–38]. Conversely, there is a need of a study to explicit these phytocompounds against a target as obstacle. Hence four reference compounds (kaempferol-3-monoglucoside, Delphinidin-3,5-diglucoside, Malvidin-3-*O*-glucoside and Quercetin) were elected to endure docking studies, comparable with four experimented phytochemicals such as (Z)-9,17-octadecadienal, 2,3-dihydro-3,5-dihydroxy-6-methyl-4H-pyran-4-one, n-decanoic acid and n-hexadecanoic acid. These compounds were preferred by considering their retention time and medicinal assets. The phytocompounds had the potential to dock with the target proteins and their interaction details are listed in Table 2 and illustrated in Fig. 2. The target protein Monoamine oxidase (MAO) A was counteracted by three and (MAO) B with two reference compounds (Fig. 3), where kaempferol-3-monoglucoside exhibited with a least score of −13.90/−12.95 kcal/mol. Binding studies was competitive among all the four test compounds the major compound (*Z*)-9,17-octadecadienal obtained from the gas chromatogram assay with a high retention time (32.99) exhibited a minimum binding affinity energy values of −6.5/−7.71 kcal/mol against both the isoforms of monoamine oxidase (MAO) A and B. n-Hexadecanoic acid commonly known as palmitic acid conferred resistant against monoamine oxidase (MAO) B as depicted in Fig. 3c with a least docking score of −10.5001 kcal/mol. The active site associated with both the isoforms of monoamine oxidase is composed of amino acid residues, such as TYR 69, TYR 197, PHE 208, GLU 216, TYR 407, PHE 352, TYR 444 (MAO) A and PHE 103, PRO 104, TRP 119, LEU 167, PHE 168, LEU 171, ILE 199, ILE 316 and TYR 326 (MAO) B [34, 39, 40]. The phytocompounds quercetin and 2,3-dihydro-3,5-dihydroxy-6-methyl-4H-pyran-4-one interacted with the active site of (MAO) A with the amino acid residue PHE 208. The study investigated diversity in the amino acid residues distinct from the active sites of both MAO A (ALA 68, GLN 443, GLN 66, MET 445, TYR 69 and ASN 181) and MAO B (LYS 296, TYR 60, GLY 434, MET 486, ILE 197, TYR 435 and CYS 172) (Table 3).

**Table 2:** Hit list of the ligands with the target protein MAO-A

S. no	Compound	Binding score energy value (Kcal/mol)	No. of hydrogen bonds	Hydrogen bond length	Interacting amino acid residue
1.	Name: kaempferol-monoglucoside Formula: C_21_H_20_O_1_ MW: 448.3769 Pub Chem ID: 5282102	−13.9077	7	2.05 2.19 2.06 1.57 2.41 1.88 2.02	ALA 68 GLN443 GLN66 GLN443 MET445 TYR69 ASN181
2.	Name: Quercetin Formula: C_20_H_40_O MW: 296.531 Pub Chem ID: 5280343	−11.4246	2	2.17 2.16	ASN 181 PHE 208
3.	Name: Malvidin-3-0-glucoside Formula: C_23_H_2 5_O_12_ MW: 493.4374 Pub Chem ID: 443652	−7.64073	3	2.32 2.25 2.15	ALA 68 TYR 69 GLN215
4.	Name: Delphinidin-3,5-diglucoside Formula: C_27_H_31_O_17_ MW: 627.52484 Pub Chem ID: 10100906	–	–	–	–
5.	Name: 9,17 Octadecadienal,(Z)- Formula: C_18_H_32_O MW: 264 Pub Chem ID: 5365667	−6.59394	2	1.86 1.87	ALA 68 TYR 69
6.	Name: 4H-Pyran-4-one, 2,3-dihydro-3,5-dihydroxy-6-methyl- Formula: C_6_H_8_O_4_ MW: 144 Pub Chem ID: 119838	−6.21223	1	1.72	PHE208
7.	Name: n-Hexadecanoic acid Formula: C_16_H_32_O_2_ MW: 256 Pub Chem ID: 985	−5.06337	3	2.21 2.05 2.10	ALA 68 ALA 68 MET 445
	8. Name: n-Decanoic acid Formula: C_10_H_20_O_2_ MW: 172 Pub Chem ID: 2969	−4.03859	3	2.13 2.16 1.96	MET 445 TYR 69 ALA 68

**Table 3:** Hit list of the ligands with the target protein MAO-B

S. no	Compound	Binding score energy value (Kcal/mol)	No. of hydrogen bonds	Hydrogen bond length	Interacting amino acid residue
1.	Name: kaempferol-monoglucoside Formula: C_21_H_20_O_1_ MW: 448.3769 Pub Chem ID: 5282102	−12.9503	3	2.25 1.76 1.73	LYS 296 TYR 60 GLY 434
2.	Name: Quercetin Formula: C_20_H_40_O MW: 296.531 Pub Chem ID: 5280343	−10.637	1	1.88	GLY 434
3.	Name: Malvidin-3-0-glucoside Formula: C_23_H_2 5_O_12_ MW: 493.4374 Pub Chem ID: 443652	–	–	–	–
4.	Name: Delphinidin-3,5-diglucoside Formula: C_27_H_31_O_17_ MW: 627.52484 Pub Chem ID: 10100906	–	–	–	–
5.	Name: n-Hexadecanoic acid Formula: C_16_H_32_O_2_ MW: 256 Pub Chem ID: 985	−10.5001	1	2.43	TYR 60
6.	Name: 9,17 Octadecadienal,(Z)- Formula: C_18_H_32_O MW: 264 Pub Chem ID: 5365667	−7.71444	1	2.01	MET 436
7.	Name: 4H-Pyran-4-one, 2,3-dihydro-3,5-dihydroxy-6-methyl- Formula: C_6_H_8_O_4_ MW: 144 Pub Chem ID: 119838	−5.3433	2	2.04 2.00	ILE 198 TYR 435
	8. Name: n-Decanoic acid Formula: C_10_H_20_O_2_ MW: 172 Pub Chem ID: 2969	−4.29637	1	1.85	CYS 172

## Conclusion

The present study provides an evidence for the extricated phytocompounds from the plant *C. ternatea* as new potent and selective hMAO-inhibitors. The results hoists two compounds, (*Z*)-9,17-octadecadienal and n-hexadecanoic acid as potential lead molecules for developing novel selective MAO-A inhibitors which can confer herbal remedy in the treatment of psychiatric disorders such as depression, anxiety, and also cognitive impairments in Alzheimer’s and Parkinson’s Diseases. The in silico assay endorses the reference phyto compound Kaempferol–monoglucoside as an established brain drug. Further the study commences amino acid residues which are distinct from the residues pertaining to the active sites of monoamine Oxidase A and B. The efficacy of these potent phytocompounds can be further elucidated with clinical studies.

## Experimental Section

### Chemicals

All chemicals used were of analytical grade purchased from, sigma Aldrich Ltd.

### Plant Collection and Identification

The healthy plant samples were collected from the premises of Bishop Heber College, Tiruchirappalli, India. The roots from the whole plant was segregated, cleaned and allowed to dry under the shade. The identification and voucher specimen number AAM 001 of the plant was sorted out and deposited at the Rapinat Herbarium and Centre for Molecular Systematic (St. Joseph’s, College Tiruchirappalli, India). The authentication of the plant as *C. ternatea* L. was validated by Dr. S. John Britto (Director).

### Preparation of Plant Extract

The shade dried powdered root sample (100 gm) was extracted with 250 mL of ethanol in a Soxhlet apparatus for 72 h. The plant extract yielded was filtered and evaporated to dryness which was further used for analysis.

### Gas Chromatography–Mass Spectrometry Analysis

GC–MS analysis of the plant extract was performed using a Perkin–Elmer Clarus 500 system comprising an AOC-20i auto-sampler. The Gas Chromatograph is interfaced to a Mass Spectrometer (GC–MS) equipped with a column (Id: 250 µm,) Elite-5MS (5 % diphenyl/95 % dimethyl poly siloxane) fused extended to a length of (30 m). For GC–MS detection, an electron ionization system was operated in electron impact mode with ionization energy of 70 eV. Helium gas (99.999 %) was used as a carrier gas at a constant flow rate of 1 ml/min, and a sample injection volume of 1.6 μl was employed (a split ratio of 10:1). The injector temperature was maintained at 280 °C, the ion-source temperature was 200 °C, the oven temperature was programmed from 60 °C (isothermal for 8 min) which, increased to 200 °C, for 5 min at 7 °C to 280 °C, ending with 280 °C (isothermal) for 15 min. Mass spectra were taken at 70 eV at a scan interval of 0.5 s and fragments from 45 to 450 Da. The relative percentage amount of each component was calculated by comparing its average peak area to the total areas. The mass-detector used in this analysis was Turbo-Mass Gold-Perkin-Elmer, with the software Turbo-Mass ver-5.2 to handle the mass spectra and chromatograms.

### Identification of Phytocomponents

Interpretation on mass-spectrum GC–MS was conducted using the database of National Institute Standard and Technology (NIST) having more than 62000 patterns. The spectrum of the unknown components was compared with the spectrum of known components stored in the NIST library. The name, molecular weight, and structure of the components of the test materials were ascertained.

### Docking Analysis

Molecular docking studies were carried to identify the binding affinities and interaction between the inhibitors and the target proteins (Mono Amine Oxidase) MAO-A and MAO-B using Glide software (Schrodinger Inc. U.S.A.- Maestro version 10.2). Grid-based Ligand Docking with Energetic (Glide) is one of the most accurate docking tool available for ligand–protein, protein–protein binding studies. Glide was found to produce least number of inaccurate poses and 85 % of Glides binding models had an RMSD of 1.4 A^°^ or less from native co-crystallized structures.

### Preparation of Ligands

**Fig. 4:**
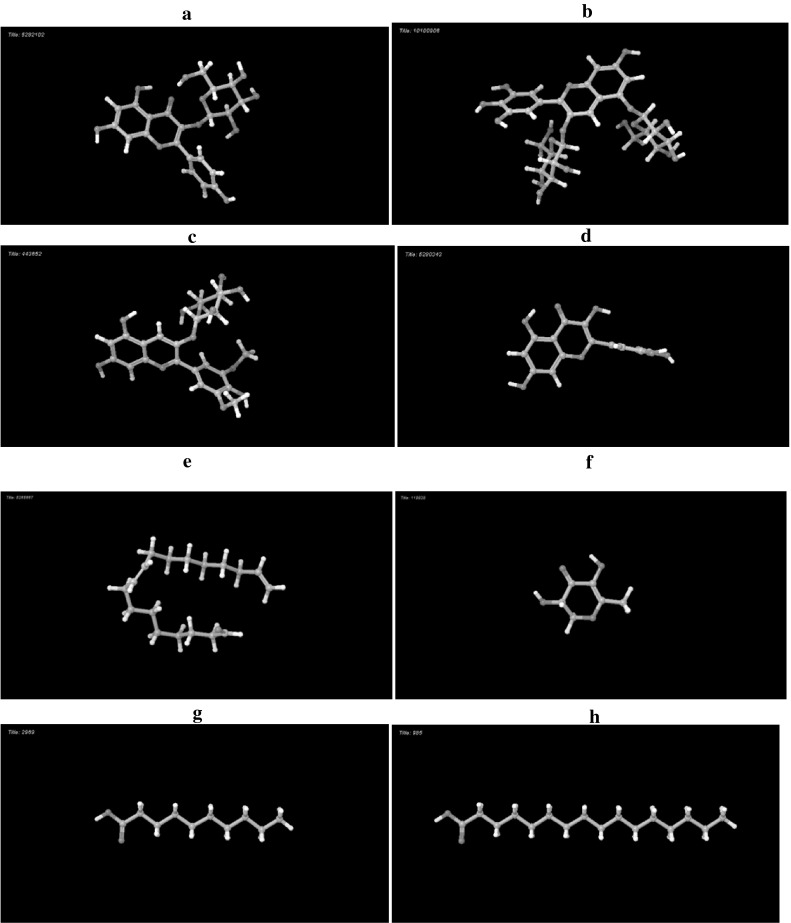
3 D Structures of the phytochemicals as ligands. **a** kaempferol-3-monoglucoside (CID: 5282102). **b** Delphinidin-3,5-diglucoside (CID:10100906). **c** Malvidin-3-0-glucoside(CID: 443652). **d** Quercetin (CID: 5280343). **e** d.9,17-Octadecadienal, (Z)- (CID: 5365667). **f**. 4H-Pyran-4-one, 2,3-dihydro-3,5-dihydroxy-6-methyl (CID:119838). **g** n-Decanoic acid (CID: 2969). **h** n-Hexadecanoic acid (CID: 985)

The 3-dimensional structures of the phytocompounds considered as ligands were retrieved and downloaded (Fig. 4a–h) as mol files from the site of Pub Chem. The molecules were processed using the LigPrep tool from Schrodinger to obtain the perfect conformation by the addition or removal of hydrogen atoms with respect to the OPLS_2005 force field.

### Preparation of Protein Target

 The target proteins Mono Amine Oxidase MAO-A and MAO-B were retrieved from Protein Data Bank (PDB). Water molecules were removed and a single chain was selected between two chains. Generally, all waters (except those coordinated to metals) are deleted, but water that connects between the ligand and the protein are sometimes retained. Problems in the PDB protein structure were repaired by adjusting the protein, metal ions, and cofactors. The structure forming bonds from the ligand or a cofactor to a protein metal were deleted by adjusting the ligand bond orders and formal charges. The minimization was done to restrain the input protein coordinates by a selected RMSD tolerance.

### GLIDE/Ligand Docking

Grid generated output file was uploaded as an input for Ligand docking against protein prepared targets in GLIDE. SP (Standard Precision) mode was adopted. Flexible docking mode was selected.
